# Non-linear association between diabetes mellitus and pulmonary function: a population-based study

**DOI:** 10.1186/s12931-020-01538-2

**Published:** 2020-11-04

**Authors:** Rui-Heng Zhang, Jian-Bo Zhou, Yao-Hua Cai, Lin-Ping Shu, Rafael Simó, Albert Lecube

**Affiliations:** 1 Beijing Tongren Hospital, Capital Medical University, Beijing, China; 2 Department of Endocrinology, Beijing Tongren Hospital, Capital Medical University, Beijing, China; 3 Endocrinology and Nutrition Department, Hospital Universitari Vall D’Hebron, Diabetes and Metabolism Research Unit, Vall D’Hebron Institut de Recerca (VHIR), Universitat Aut`Onoma de Barcelona, Barcelona, Catalonia Spain; 4 Endocrinology and Nutrition Department, Hospital Universitari Arnau de Vilanova, Obesity, Diabetes and Metabolism Research Group (ODIM), Institut de Recerca Biom`Edica de Lleida (IRBLleida), Universitat de Lleida, Lleida, Catalonia Spain

**Keywords:** Diabetes mellitus, Pulmonary function, NHANES

## Abstract

**Background:**

There is increasing evidence that the lung is a target organ of diabetes. This study aimed to examine in detail the association between diabetes mellitus and pulmonary function using a national cohort. We also aimed to explore the non-linear association between pulmonary function and blood glucose, insulin resistance, and C-reactive protein (CRP).

**Methods:**

A total of 30,442 participants from the National Health and Nutrition Examination Survey from the period between 2007 and 2012 were included. The cross-sectional association between diabetes mellitus and pulmonary function was assessed using multiple linear regression. Where there was evidence of non-linearity, we applied a restricted cubic spline with three knots to explore the non-linear association. Partial mediation analysis was performed to evaluate the underlying mechanism. All analyses were weighted to represent the US population and to account for the intricate survey design.

**Results:**

A total of 8584 people were included in the final study population. We found that diabetes was significantly associated with reduced forced expiratory volume in one second (FEV_1_) and forced vital capacity. We further found L-shaped associations between hemoglobin A1c (HbA1c) and pulmonary function. There was a negative association between HbA1c and FEV_1_ in diabetes participants with good glucose control (HbA1c < 7.0%), but not in patients with poor glucose control. A non-linear association was also found with fasting plasma glucose, 2 h-plasma glucose after oral glucose tolerance test, insulin resistance, and CRP. Finally, we found that diabetes duration did not affect pulmonary function, and the deleterious effect of diabetes on pulmonary function was mediated by hyperglycemia, insulin resistance, low-grade chronic inflammation (CRP), and obesity.

**Conclusions:**

Diabetes mellitus is non-linearly associated with pulmonary function. Our finding of a negative association between HbA1c and FEV_1_ in diabetes patients with good glucose control but not in patients with poor glucose control indicates that a stricter glycemic target should be applied to diabetic patients to improve pulmonary function. Given, the cross-sectional nature of this research, a longitudinal study is still needed to validate our findings.

## Background

The global number of adults with diabetes is expected to surpass 700 million by 2025 [1]. Complications include microvascular and macrovascular conditions, such as retinopathy, nephropathy, neuropathy, cardiovascular, and peripheral vascular diseases [2]. There is now increasing evidence indicating that the lung is also a target organ of diabetes [3]. As reported by van den Borst et al. [4], diabetes is associated with a significantly decreased forced expiratory volume in 1 s (FEV_1_%) and forced vital capacity (FVC%) predicted value with a preserved FEV_1_/FVC ratio. However, the pathophysiological mechanisms underlying the development of this deleterious effect of type 2 diabetes mellitus (T2DM) and its aggravation are still mostly unclear, with conflicting evidence. Among the well-recognized mechanisms, the metabolic pathways related to insulin resistance and low-grade chronic inflammation, inherent in T2DM pathophysiology, have been emphasized [5, 6]. In addition, the effect of metabolic control on pulmonary function is also controversial. For example, in the Hispanic Community Health Study/Study of Latinos, FEV_1_ was not corrected with hemoglobin A1c (HbA1c) in the diabetic population [7]. In contrast, in the Sweet Breath Study, 60 subjects received a 3-month intensified treatment program for diabetes. The short-term benefit of glycemic control on pulmonary function was profound for good responders (defined as an HbA1c decrease > 0.5% after treatment), who exhibited a significant improvement in spirometry values between baseline and the end of the study [8].

These different pieces of evidence indicate a complex association between diabetes and pulmonary function, which warrants this study. We aimed to examine in detail the association of diabetes with pulmonary function using data from the 2007–2012 National Health and Nutrition Examination Survey (NHANES), a US national cohort.

## Methods

### Data source and participants

The data analyzed in this study came from the NHANES database. When using the NHANES data, we chose the period from 2007 through to 2012 for inclusion because of the consistent pulmonary function testing used during that period. The NHANES, which is described in detail elsewhere [9], is a multistage probability sample of the non-institutionalized US population and allows estimates that represent the US population. A total of 30,442 participants were included in the NHANES from 2007 to 2012. We excluded individuals: (1) aged < 18 years (11,823 participants), (2) with missing information on diabetes mellitus (questionnaire, HbA1c, fasting plasma glucose [FPG], or 2 h-plasma glucose after oral glucose tolerance test (OGTT): two participants), (3) ethnic minorities (3611 participants), (4) with missing pulmonary function test results (3657 participants), (5) with missing pulmonary function test results that met the American Thoracic Society data collection standards (1480 participants), (6) who were pregnant at examination (106 participants), and (7) with pulmonary comorbidities (including asthma, chronic bronchitis, and emphysema: 1179 participants). Ethnic minorities included races other than Mexican American, Non-Hispanic White, or Non-Hispanic Black and they were excluded because relevant spirometric reference values are often not available. In total, 8584 participants were included in this study. No participants had undergone chest or abdominal surgery within 3 months.

### Exposures and outcomes

The diagnosis of diabetes was based on the question: “Other than during pregnancy, have you ever been told by a doctor or health professional that you have diabetes or sugar diabetes?” Diabetes duration was based on the question: “How old were you when a doctor or other health professional first told you that you had diabetes or sugar diabetes?”. Also, diabetes was diagnosed according to the American Diabetes Association (ADA) standard [10], which is a FPG ≥ 126 mg/dL (7.0 mmol/L) or 2-h plasma glucose after the OGTT test (2 h-PG) ≥ 200 mg/dL (11.1 mmol/L) or A1C ≥ 6.5%. When diabetes was diagnosed by the ADA standard with a denied response on the questionnaire, we assumed a diabetes duration of 0 years. Prediabetes was further diagnosed among participants without diabetes according to the ADA standard [11], which is an FPG ≥ 100 mg/dL (5.6 mmol/L) and ≤ 125 mg/dL (6.9 mmol/L) or a 2 h-PG ≥ 140 mg/dL (7.8 mmol/L) and ≤ 199 mg/dL (11.0 mmol/L) or an HbA1c ≥ 5.7% and ≤ 6.4%. Pulmonary comorbidities (including asthma, chronic bronchitis, and emphysema) were ascertained by self-report.

During the NHANES between 2007 and 2012, spirometry was offered to participants aged 6 to 79 years with the exclusion of participants with the following: current chest pain; a physical problem with forceful expiration; use of supplemental oxygen; recent surgery of the eye, chest, or abdomen; recent heart attack, stroke, tuberculosis exposure, or coughing up of blood; and history of a detached retina, collapsed lung, or aneurysm. Similar dry-rolling seal volume spirometers (Ohio 822/827; Ohio Medical Instrument Company, Cincinnati, OH, USA) and similar protocols were used for conducting spirometry. Participants were asked to provide three acceptable maneuvers. For the purposes of this study, we only used pre-bronchodilator spirometry data with quality A (exceeds American Thoracic Society data collection standards or B (meets American Thoracic Society data collection standards). The predicted pulmonary function (FEV_1_, FVC) was calculated according to the NHANES III equations [12].

### Covariables

The definitions and methods used for other baseline measurements (age, sex, ethnicity, height, waist circumference [WC], body mass index [BMI]) have been described in detail elsewhere [9]. We defined “current smoker” as a participant who smoked every day. Insulin resistance was assessed by the homeostasis model of assessment for insulin resistance index (HOMA-IR), which was calculated as fasting plasma insulin (mU/L) × FPG (mmol/L)/22.5 [13]. Physical activity was assessed by metabolic equivalent scores (MET) [9] = vigorous work-related activity*8 + moderate work-related activity *4 + walking or bicycling for transportation*4 + vigorous leisure-time physical activity*8 + moderate leisure-time physical activity*4.

### Statistical analysis

A student’s t-test determined between-group differences in cross-sectional characteristics for continuous data with a normal distribution and non-normal distribution data after log-transformation. The chi-squared test was used for dichotomous and categorical data. Multiple logistic regression models were applied to assess the multivariable associations between diabetes and pulmonary function parameters. Where there was evidence of nonlinearity in Kernel-weighted local polynomial smoothing, a two-line piecewise linear model with a single change point was estimated by trying all possible values for the change point and choosing the value with the highest likelihood. Then, we applied a restricted cubic spline with three knots to explore the non-linear association. The first and last knots were placed at the 1%- and 99%-point of the examined parameters, respectively, and the middle knot was placed at the point chosen by the two-line piecewise linear model. It should be emphasized that we included all participants in the analysis of nonlinearity. Partial mediation was assessed using the percent mediation calculated as the relative change in FEV_1_ and FVC associated with the occurrence of diabetes between the baseline model and the adjusted model (also called percent change [PC]) [14].

A two-sided p-value < 0.05 was considered statistically significant. All analyses were weighted to represent the US population and to account for the intricate survey design and performed in STATA (15.0) STATA Corporation, College Station, TX, USA).

## Results

### Cross-sectional characteristics of the participants

The main clinical and pulmonary data of the 8584 participants who were eligible for this cross-sectional study according to the presence of glucose abnormalities are displayed in Table 1. The weighted proportions of prediabetes and diabetes were 29.5% and 10.1%, respectively. Compared to participants with normal glucose metabolism, those with prediabetes and diabetes were significantly older and more likely to be male, Mexican American, and non-Hispanic Black, with a lower educational level, physically inactive, and with a greater average BMI. The ratio of current smokers did not differ between the groups. Regarding pulmonary function, participants with prediabetes and diabetes showed significantly lower FEV_1_ and FVC measurements than the controls. This progressive association persisted after adjustment for confounding variables such as age, sex, race, educational level, physical activity (MET score), smoking status, BMI and WC (Additional file 1: Table S1). Finally, once supplementary adjustments for C-reactive protein (CRP) and insulin resistance were performed, participants with diabetes continued to show significantly decreased FEV_1_ and FVC values, reinforcing the potential role of other mechanisms underlying this negative association.

**Table 1:** Cross-sectional characteristics of participants

Characteristic	Glucose normal (n = 4689)	Prediabetes (n = 2657)	Diabetes (n = 1238)	P-trend
Weighted proportion %	60.4 (0.8)	29.5 (0.6)	10.1 (0.5)	
Age (year)	39.8 (0.5)	47.7 (0.5)	56.5 (0.5)	< 0.001
Male (%)	48.1 (0.8)	53.1 (1.0)	52.7 (2.0)	< 0.001
Ethnicity %				
Mexican American	9.5 (1.2)	10.4 (1.4)	12.6 (1.9)	0.007
Non-Hispanic White	80.4 (1.7)	76.7 (2.1)	69.4 (3.2)	< 0.001
Non-Hispanic Black	10.1 (1.1)	12.9 (1.3)	18.0 (2.3)	< 0.001
BMI	27.2 (0.1)	29.8 (0.2)	33.4 (0.3)	< 0.001
Height (cm)	170.1 (0.2)	170.0 (0.2)	168.6 (0.4)	0.008
Waist circumference	93.8 (0.4)	101.6 (0.4)	111.1 (0.6)	< 0.001
Eucation level (%)	32.8 (1.6)	26.2 (1.6)	19.5 (2.1)	< 0.001
Current smoker %	16.5 (1.0)	19.6 (1.1)	15.1 (1.0)	0.39
MET scores	600 [160–1440]	480 [80–1200]	180 [0–720]	< 0.001
FPG	5.10 (0.01)	5.77 (0.02)	8.19 (0.15)	< 0.001
2 h-PG	5.16 (0.04)	6.57 (0.05)	12.6 (0.35)	< 0.001
HbA1c %	5.22 (0.01)	5.65 (0.01)	7.12 (0.06)	< 0.001
HOMA-IR	1.7 [1.1–2.6]	3.0 [1.8–4.6]	4.8 [2.7–8.5]	< 0.001
Serum insulin (uU/mL)	7.7 [5.1–11.2]	11.7 [7.4–17.3]	14.6 [8.3–23.2]	< 0.001
C-Reactive Protein (mg/dL)	0.13 [0.05–0.29]	0.21 [0.09–0.48]	0.31 [0.12–0.71]	< 0.001
Diabetes duration (years)			3 [0–10]	
FEV_1_%predicted	98.9 (0.3)	95.1 (0.5)	90.8 (0.7)	< 0.001
FVC %predicted	101.3 (0.3)	98.6 (0.4)	92.6 (0.6)	< 0.001
FEV_1_/FVC ratio	79.4 (0.2)	76.5 (0.2)	76.5 (0.3)	< 0.001

Data were weighted estimates, and expressed as mean (standard error) or median [percentile 25 -percentile 75] when appropriate. *Education level* percentage of participants completed college graduate or above, *FPG* fasting plasma glucose, *2 h-PG* plasma glucose 2 h after OGTT, *HOMA-IR* homeostasis model of assessment for insulin resistance index, *MET score* metabolic equivalent scores *p < 0.05 compared to glucose normal

### Non-linear association between diabetes and pulmonary function

**Fig. 1:**
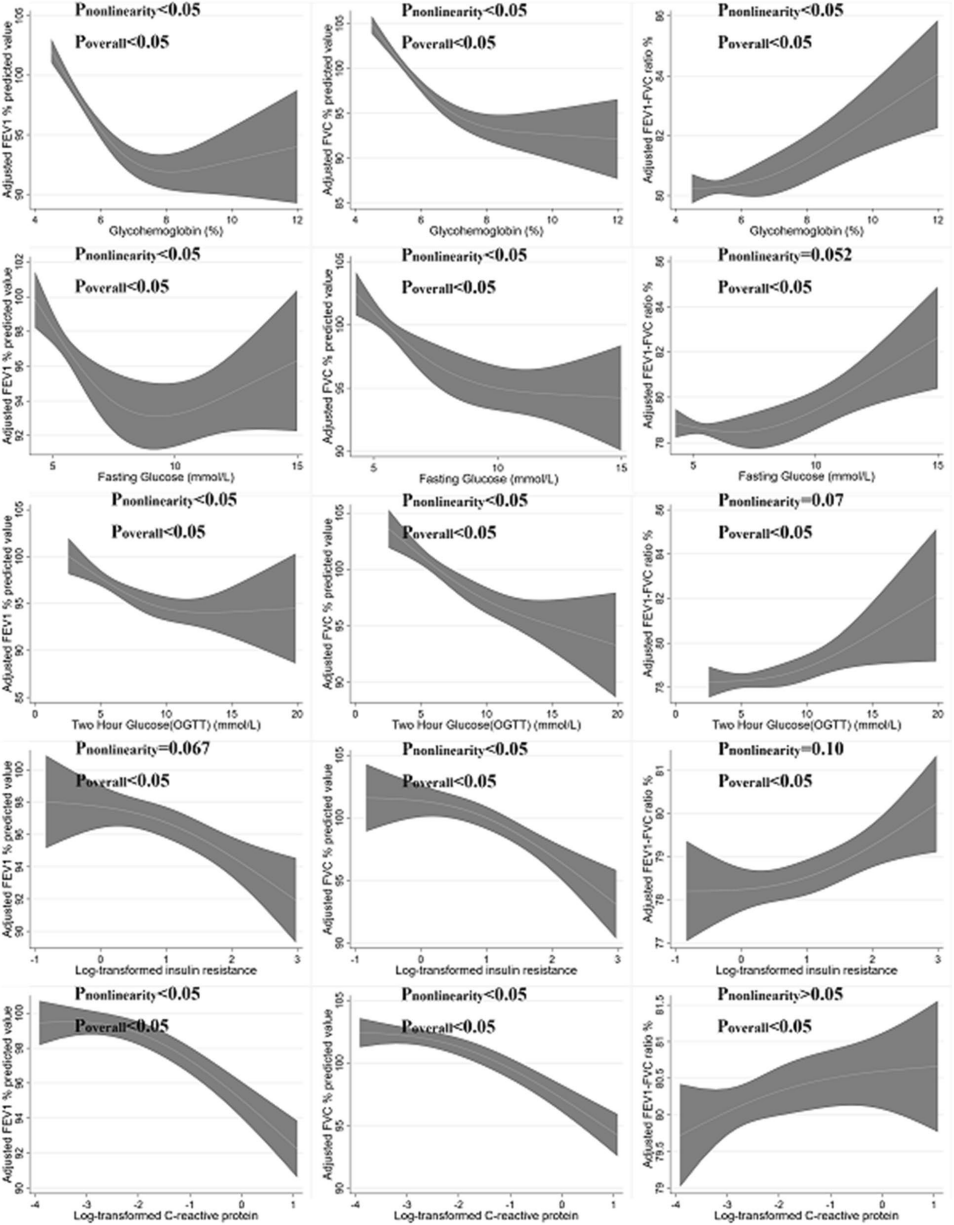
Non-linear association between plasma glucose, insulin resistance, CRP and pulmonary function in participants without pulmonary comorbidities. Data were weighted estimates. The shadow area represents a 95% confidence interval. P_non-linear_ was estimated by a two-line piecewise linear model. The model was adjusted for age, sex, race, education, smoking, physical activity, BMI and waist circumference

After adjustment for age, sex, race, education, smoking, physical activity, BMI, and WC, associations between HbA1c and FEV_1_, as well as FVC, were L-shaped (Fig. 1). The change points for FEV_1_ and FVC were estimated to be approximately 7%. When participants with diabetes were classified according to their HbA1c value, the negative association between HbA1c and FEV_1_ [− 7.23 (− 11.17 to − 3.29)] and FVC [− 5.31 (− 8.65 to − 1.97)] existed only in those with good metabolic control (HbA1c < 7.0%) (Additional file 1: Table S2). A similar non-linear association was also found in FPG as well as 2 h-PG.

Furthermore, we found a non-linear association between insulin resistance and pulmonary function in FVC and borderline significance in FEV_1_, but not in FEV_1_/FVC ratio. The non-linear association between CRP and pulmonary function also existed with a change point estimated at 0.14 mg/dL.

### Mediation of the effect of diabetes on pulmonary function

Diabetes duration may have been a major confounding factor. However, as shown in Additional file 1: Table S3, both undiagnosed (diagnosed by ADA standard with the denied response on the questionnaire) and diagnosed diabetes (doctor or other health professional diagnosed diabetes) patients have similar pulmonary function. Similarly, diabetes duration (years) was not associated with pulmonary function (FEV_1_ coefficient = 0.07 [− 0.13, 0.27]; FVC coefficient = − 0.01 [− 0.18, 0.16]; FEV_1_/FVC coefficient = 0.06 [− 0.01, 0.13]). Thus, we further performed mediation analysis. Table 2 presents a summary of the salient results for the subset of factors mediating the association between diabetes and either FEV_1_ or FVC. HbA1c subgroups (< and ≥ 7.0) were created because of the nonlinearity of HbA1c. The effect of well-controlled diabetes was an attenuation (i.e., mediation) of approximately 40% for FEV_1_ and FVC, with adjustment for other potential mediating risk factors one at a time. Plasma glucose was the major mediator. Other mediators included insulin resistance, CRP, and obesity. Similar results were found in patients with HbA1c ≥ 7.0. As the negative association between HbA1c and pulmonary function existed only in those with good metabolic control, both plasma glucose and insulin resistance were major mediators in patients with HbA1c ≥ 7.0.

**Table 2:** Coefficient and 95% confidence intervals for the effect of diabetes on the pulmonary function

	DM with Hba1c < 7				DM with Hba1c > = 7			
	FEV_1_	PC	FVC	PC	FEV_1_	PC	FVC	PC
Model 1 adjusted for	− 6.54 [− 8.17, − 4.91]		− 7.21 [− 8.94, − 5.47]		− 6.68 [− 8.83, − 4.53]		− 9.17 [− 11.47, − 6.87]	
Additionally adjusted for								
HbA1c	− 4.17 [− 6.03, − 2.32]	36.3%	− 4.71 [− 6.66, − 2.77]	34.6%	− 4.88 [− 8.35, − 1.41]	31.1%	− 7.20 [− 10.73, − 3.67]	24.7%
FPG	− 4.62 [− 8.16, − 1.10]	32.5%	− 5.89 [− 9.22, − 2.56]	21.2%	− 4.83 [− 9.71, 0.06]	24.2%	− 7.22 [− 12.23, − 2.21]	17.1%
2 h-PG	− 3.19 [− 8.02, 1.65]	40.3%	− 3.55 [− 8.10, 1.01]	43.2%	IO	IO	IO	IO
log (HOMA-IR)	− 5.81 [− 8.77, − 2.85]	15.4%	− 5.33 [− 8.12, − 2.54]	26.9%	− 5.33 [− 8.06, − 2.59]	19.7%	− 6.83 [− 9.47, − 4.19]	23.5%
BMI	− 6.01 [− 7.91, − 4.30]	6.7%	− 6.39 [− 7.97, − 4.81]	11.5%	− 6.12[ − 8.18, − 4.07]	8.6%	− 8.24 [− 10.10, − 6.38]	10.4%
waist circumference	− 5.62 [− 7.41, − 3.83]	15.5%	− 6.06 [− 7.67, − 4.45]	17.6%	− 5.21 [− 7.25, − 3.17]	20.6%	− 7.55 [− 9.42, − 5.67]	17.6%
log (C-reactive protein)	− 4.89 [− 6.70, − 3.08]	12.7%	− 5.97 [− 7.75, − 4.18]	12.0%	− 4.92 [− 7.69, − 2.15]	24.8%	− 8.46 [− 10.85, − 6.06]	11.3%

*PC* Percentage mediation, *IO* insufficient observation, *FPG* fasting plasma glucose, *2 h-PG* plasma glucose 2 h after OGTT, *HOMA-IR* homeostasis model of assessment for insulin resistance index

Model 1 adjusted age, race, sex, education, and METs

### Discussion

To our knowledge, this is the first study to demonstrate the non-linear association between metabolic control (HbA1c, FPG, and 2 h-PG) and pulmonary function. Furthermore, we found that a non-linear association also existed between HOMA-IR, CRP, and pulmonary function. We validated the hypothesis that the negative association of HbA1c, FEV_1_, and FVC existed only in participants with diabetes and good glucose control (HbA1c < 7.0%), indicating that pulmonary function may benefit from a stricter glycemic target. Finally, we found that diabetes duration did not affect pulmonary function, and the deleterious effect of diabetes on pulmonary function was mediated by hyperglycemia, insulin resistance, low-grade chronic inflammation (CRP), and obesity.

There is now increasing evidence indicating that the lung is also target organ of diabetes [3]. The extent of the deleterious effect of diabetes on pulmonary function has probably been overestimated because most cross-sectional studies did not provide data after adjustments for confounding factors. For example, obesity and current smokers were more common in type 2 diabetes populations, thus becoming the most critical covariables. A study by Giovannelli et al. revealed that the unadjusted FVC in diabetes participants was − 6.9% (range: − 9.1 to − 4.7) lower than in non-diabetes participants, and adjustment for smoking and BMI attenuated this association − 4.4% (range: − 6.7 to − 2.2) [15]. Similar results were also observed in the study by Hickson et al. [16]. Our study further confirmed this conclusion in a national cohort, supporting that the negative relationship between diabetes and pulmonary function, although overestimated, persisted after a strict adjustment for the main available confounding factors.

As plasma glucose has a continuous association with diabetic complications, one would expect a consistent and continuous negative association between prediabetes, diabetes, and pulmonary function. However, this is not in line with reality. The L-shaped association between HbA1c, FPG, and 2 h-PG and the two main parameters of pulmonary function (FEV_1_ and FVC) means that a negative association exists in those subjects with normal glucose, when prediabetes is diagnosed, and in those patients with diabetes that achieve better metabolic control. Research has previously demonstrated in a large cross-sectional study that the deleterious effect of type 2 diabetes on pulmonary function begins in the prediabetes stage and that it is related to metabolic control [17, 18]. Our current data reinforces this continuous decrement of FEV_1_ and FVC values from normoglycemia to diabetes and reveal the harmful effect of hyperglycemia on pulmonary function onset before the diagnosis threshold of diabetes.

The L-shaped association between plasma glucose and the two main parameters of pulmonary function (FEV_1_ and FVC) indicates that a negative association exists in those subjects with normal glucose, when prediabetes is diagnosed, and in those patients with diabetes who achieve better metabolic control. Prediabetes shows an estimated prevalence of 38.0% in the overall 2011–2012 population in adults in the United States [37]. This intermediate metabolic state between normal glucose metabolism and type 2 diabetes is associated with a higher incidence of diabetic microangiopathy as well as increased cardiovascular disease and all-cause mortality [38]. However, the “saturation” phenomenon in “diabetic lung” indicates a distinct evolution between lung impairment and classical complications in T2DM. Similarly, a large cross-sectional study with 4459 participants showed that the deleterious effect of T2DM on pulmonary function begins in the prediabetes stage, and that it is related to metabolic control [18]. Our current data reinforce this continuous decrement of FEV^1^ and FVC values from normoglycemia to diabetes and reveal the harmful effect of hyperglycemia on pulmonary function onset before the diagnosis threshold of diabetes.

The impaired pulmonary function in diabetes participants exhibited reduced FEV_1_ and FVC with a preserved FEV_1_/FVC, namely a restrictive pattern [19]. However, diabetes has also been associated with reduced diffusing lung capacity for carbon monoxide (DLCO) and DLCO/VA (interpreted as the efficiency of alveolar transfer of carbon monoxide) [4], indicating a more complex relationship than chest wall restriction (such as obesity) [20]. Non-enzymatic glycation of lung collagen and elastin has been suggested as a potential mechanism involved in increased matrix stiffness [3]. Similarly, the lung alveolar-capillary network constitutes the largest microvascular bed in humans, making it susceptible to diabetic microangiopathy. In Chance’s study, the authors found that decreased DLCO in diabetes participants was proportionally related to decreased pulmonary blood flow both at rest and at 90% peak workload [21]. In addition, lung biopsy revealed the thickness of the pulmonary epithelial and endothelial basal lamina in patients with diabetes [22].

The role of insulin resistance and low-grade chronic inflammation (CRP) in initiating lung abnormalities deserves more attention. Cross-sectional data of 922 nondiabetic participants in the Normative Aging Study found that fasting insulin and insulin resistance were negatively correlated with FVC and FEV_1_ [23]. In the Strong Heart Study, this negative correlation extended to 1184 diabetes participants [24]. Similarly, in a representative sample of adolescents from the US in the 2007–2010 NHANES, HOMA-IR was negatively associated with FEV_1_ and FVC [25]. Our team used 10-year follow-up data from the Coronary Artery Risk Development in Young Adults (CARDIA) cohort, and found that a higher HOMA-IR trajectory was associated with abnormal spirometry performance that is characterized by decreased FEV_1_ and FVC but a preserved FEV_1_/FVC ratio from childhood to adulthood (unpublished data). Two of the advocated mechanisms linking insulin resistance and pulmonary dysfunction are the impairment of skeletal muscle strength due to a reduction in mitochondrial fitness and the powerlessness of insulin receptors located in type II alveolar epithelial cells to stimulate surfactant production [26–28]. Systemic inflammation mediated by insulin resistance may also be a contributing factor [29]. Tumor necrosis factor (TNF) receptor 1 is mainly expressed in epithelial lung cells, and the importance of TNF-α in the inflammatory processes of the lung has been demonstrated in a mouse model of acute lung inflammation [30]. In humans, soluble TNF-α receptor 1 contributes independently to FEV_1_ and FVC impairments [31]. In cross-sectional studies, a strong inverse association between CRP levels and quartiles of FEV_1_ was observed among 1131 healthy subjects [32]. Similarly, data from the British Regional Heart Study demonstrated significant inverse associations between baseline FVC and FEV_1_ and blood markers of inflammation, including CRP [33].

Our study had many strengths, including using the fact it involved a national cohort. It is also the first to demonstrate the non-linear association between plasma glucose and pulmonary function. However, elucidating the complex association between diabetes and pulmonary function is not an easy task. Some limitations of this study should be mentioned. First, although our study mainly provided evidence that diabetes was negatively and non-linearly associated with pulmonary function, a causal association cannot be demonstrated because of its cross-sectional nature. A longitudinal study is needed to validate whether dynamic changes in plasma glucose levels are negatively associated with pulmonary function. Furthermore, studies have found that reduced pulmonary function is a risk factor for developing diabetes [34–37]. Thus, the bidirectional association may be in line with reality. Third, although we used a two-line piecewise linear model to estimate a single change point, the choice of change point may also be inaccurate. However, although the inaccuracy may have slightly affected the regression coefficient, it would not have affected the non-linear conclusions. Besides, we have no data on either type of diabetes or diabetes antidiabetic therapies in our population. Based on this, therapy-mediated improvements in insulin sensitivity may be related to the L-shaped relationship described in our study.

## Conclusions

Diabetes is non-linearly associated with pulmonary function. In terms of clinical practice, based on our finding that there is a negative association between HbA1c and FEV_1_ in diabetes patients with good glucose control but not in patients with poor glucose control we recommend that a stricter glycemic target is applied to diabetic patients to improve pulmonary function. Due to the cross-sectional nature of this study, a longitudinal study is still needed to validate our findings.

## Supplementary information

**Additional file 1: Table S1.** Association of diabetes and pulmonary function. **Table S2.** Association of HbA1c and pulmonary function in diabetes participants with good and bad glucose control. **Table S3. **Cross-sectional characteristics of diabetes patients.

## Data Availability

None.

## Abbreviations

2 h-PG: 2H-plasma glucose after OGTT test
CRP: C-reactive protein
DLCO: Diffusing lung capacity for carbon monoxide
FEV_1_: Forced expiratory volume in one second
FPG: Fasting plasma glucose
FVC: Forced vital capacity
HbA1c: Hemoglobin A1c
HOMA-IR: Homeostatic model assessment of insulin resistance
MET: Metabolic equivalent scores
NHANES: National Health and Nutrition Examination Survey
OGTT: Oral glucose tolerance test
TNF: Tumor necrosis factor
