# Supplementary material for: Non-linear association between diabetes mellitus and pulmonary function: a population-based study
Source: Respir Res. 2020 Nov 4;21:292. doi: 10.1186/s12931-020-01538-2 (PMC7641838; doi:10.1186/s12931-020-01538-2)
Supplement: Supplementary file 1 — Additional file 1: Table S1. Association of diabetes and pulmonary function. Table S2. Association of HbA1c and pulmonary function in diabetes participants with good and bad glucose control. Table S3. Cross-sectional characteristics of diabetes patients. [file 12931_2020_1538_MOESM1_ESM.docx]

**Additional file 1: Table S1.** Association of diabetes and pulmonary function

|  |  | Glucose normal  (n=4689) | Prediabetes  (n=2657) | Diabetes  (n=1238) |
| --- | --- | --- | --- | --- |
| Model 1 | FEV_1_ %predicted | reference | **-2.34 [-3.33, -1.35]** | **-5.27 [-6.70, -3.84]** |
|  | FVC %predicted | reference | **-1.80 [-2.56, -1.04]** | **-6.40 [-7.83, -4.96]** |
|  | FEV_1_/FVC ratio % | reference | -0.40 [-0.82, 0.03] | **1.07 [ 0.34, 1.81]** |
| Model 2 | FEV_1_ %predicted | reference | **-2.15 [-3.49, -0.81]** | **-4.86 [-6.46, -3.26]** |
|  | FVC %predicted | reference | **-1.93 [-2.97, -0.90]** | **-6.14 [-7.91, -4.38]** |
|  | FEV_1_/FVC ratio % | reference | -0.15 [-0.70, 0.40] | **1.21 [0.34, 2.09]** |
| Model 3 | FEV_1_ %predicted | reference | **-1.95 [-3.89, -0.02]** | **-3.86 [-6.12, -1.60]** |
|  | FVC %predicted | reference | **-1.52 [-3.04, -0.00]** | **-4.85 [-6.87, -2.83]** |
|  | FEV_1_/FVC ratio % | reference | -0.44 [-1.03, 0.16] | 0.91 [-0.18, 1.99] |

Data were weighted estimates and expressed as mean [95% confidence interval].

Model 1: Multiple linear regression adjusting for age, sex, race, education level, physical activity (MET score), smoking status, BMI and waist circumference.

Model 2: model 1 + Log-transformation CRP

Model 3: model 1 + Log-transformation CRP + Log-transformation insulin resistance

**Bold**: p<0.05;

**Additional file 1: Table S2**. Association of HbA1c and pulmonary function in diabetes participants with good and bad glucose control.

|  |  | Good control (n=681) | Bad control (n=557) |
| --- | --- | --- | --- |
|  | HbA1c % | <7.0% | ≥7.0% |
| Model 1 | FEV_1_ %predicted | **-4.14 [-7.16, -1.12]** | -0.48 [-1.18, 0.21] |
|  | FVC %predicted | -2.84 [-5.75, 0.08] | -0.73 [-1.52, 0.05] |
|  | FEV_1_/FVC ratio % | -1.12 [-2.33, 0.09] | 0.25 [-0.06, 0.57] |
| Model 2 | FEV_1_ %predicted | **-3.79 [-7.37, -0.20]** | -0.41 [-1.43, 0.60] |
|  | FVC %predicted | -3.00 [-6.43, 0.42] | -0.90 [-1.97, 0.17] |
|  | FEV_1_/FVC ratio % | -0.73 [-2.07, 0.60] | **0.41 [0.01, 0.81]** |
| Model 3 | FEV_1_ %predicted | **-7.23 [-11.17, -3.29]** | -0.14 [-1.76, 1.48] |
|  | FVC %predicted | **-5.31 [-8.65, -1.97]** | -0.61 [-2.22, 1.00] |
|  | FEV_1_/FVC ratio % | -1.74 [-3.88, 0.40] | 0.41 [-0.45, 1.28] |

Data were weighted estimates and expressed as mean [95% confidence interval].

Model 1: Multiple linear regression adjusting for age, sex, race, education level, physical activity (MET score), smoking status, BMI and waist circumference.

Model 2: model 1 + Log-transformation CRP

Model 3: model 1 + Log-transformation CRP + Log-transformation HOMA-IR

**Bold**: p<0.05;

| Characteristic | Undiagnosed diabetes | diagnosed diabetes | P-value |
| --- | --- | --- | --- |
|  | (n=410) | (n=828) |  |
| Age (year) | 55.3 (0.6) | 57.1 (0.6) | 0.028 |
| Male (%) | 54.8 (3.0) | 51.5 (2.6) | 0.42 |
| Ethnicity % |  |  |  |
| Mexican American | 15.3 (2.5) | 11.2 (1.9) | 0.03 |
| Non-Hispanic White | 70.8 (4.1) | 68.7 (3.3) | 0.56 |
| Non-Hispanic Black | 13.9 (2.6) | 20.1 (2.6) | 0.02 |
| BMI | 33.2 (0.4) | 33.5 (0.4) | 0.55 |
| Eucation level (%) | 21.3 (2.6) | 18.5 (2.3) | 0.52 |
| Current smoker % | 15.3 (2.3) | 15.0 (1.1) | 0.91 |
| MET scores | 200 [0- 800] | 180 [0- 600] | 0.15 |
| HbA1c % | 6.70 (0.08) | 7.35 (0.08) | <0.001 |
| HOMA-IR | 4.8 [3.1- 8.6] | 4.7 [2.4-8.4] | 0.045 |
| Serum insulin (uU/mL) | 16.0 [10.3- 24.5] | 12.7 [7.2- 22.1] | <0.001 |
| C-Reactive Protein (mg/dL) | 0.34 [0.14- 0.82] | 0.28 [0.11- 0.61] | 0.011 |
| Diabetes duration (years) | 0 [0- 0] | 7 [3-14] | <0.001 |
| FEV_1_ %predicted | 91.5 (0.8) | 90.5 (0.4) | 0.34 |
| FVC %predicted | 93.5 (0.7) | 92.1 (0.8) | 0.16 |
| FEV_1_/FVC ratio | 76.5 (0.5) | 76.6 (0.4) | 0.9 |
| Adjusted FEV_1_ %predicted ^¶^ | reference | -0.74 [-2.85, 1.36] |  |
| Adjusted FVC %predicted^¶^ | reference | -1.35 [-3.42, 0.71] |  |
| Adjusted FEV_1_/FVC ratio^¶^ | reference | 0.42 [-0.70, 1.55] |  |

**Additional file 1: Table S3.** Cross-sectional characteristics of diabetes patients

Data were weighted estimates, and expressed as mean (standard error) or median [percentile 25 -percentile 75] when appropriate. Education level, percentage of participants completed college graduate or above; HOMA-IR, homeostasis model of assessment for insulin resistance index. MET score, metabolic equivalent scores *p<0.05 compared to glucose normal

^¶^ Adjusted for age, sex, race, education level and MET score.
